# On Pledges Exacted from the Insane
*Read at a meeting of the American Association of Medical Officers of Hospitals for the Insane, by Dr. J. M. Galt, physician to the Eastern Lunatic Asylum, Virginia.


**Published:** 1855-01-01

**Authors:** 


### ON PLEDGES EXACTED FROM THE INSANE.*

In several of his reports, Dr. Samuel B. Woodward, whilst superintendent of the Massachusetts State Lunatic Hospital, has spoken somewhat at large in relation to that particular of moral management, which he has entitled "Pledges." The older writers have also alluded to the subject: Cox does so directly; and in many cases reported by Pinel, Esquirol, and others, the principle is involved. We find various writers, too, alluding to the fact, that the insane will rarely break their word, as for example, Mr. Hill, in his work "On Lunatic Asylums." Dr. Woodward has even extended this principle of treatment to the management of the suicidal. Dr. Bell, of the McLean Asylum, however, is opposed to going so far, observing that, "Pledges, not to abuse privileges, to go out and return punctually, and the like, may be extended to a great degree. But where the pledge would cover so all-important and irreprievable a hazard as that of life, we have never trusted it; the suicide's last act often is accomplished with false representations; his whole intent is not unfrequently accompanied with great sagacity, in throwing the inexperienced and unskilled off their guard. I should not, with my experience, pay the slightest regard to any promise which they might make respecting this act." Dr. W. S. F. Browne also remarks, in his work on "Insanity and Asylums for the Insane," that Esquirol "trusted a military man, who was determined on suicide, with the means of destruction, on his pledging his honour that he would make no attempt to use them." He then goes on to say, "He passed the ordeal in safety, but not without a struggle. This *was venturing* far, perhaps, too far." Moreover, in a letter addressed to the trustees of the New York State Lunatic Asylum, of which, by a gentleman of Baltimore, describing St. Vincent's Hospital, an institution under the management of the Sisters of Charity, we find the following remarks :—"In the pledges so much spoken of in the Worcester Report, the sister places little confidence; she states, that a lunatic derides the idea of a binding promise; especially if it be of the violent type. She thinks these unhappy sufferers are generally conscious of their lamentable condition; and will ask you, when reminded of a broken pledge, 'if you were fool enough to believe a crazy person?'" So far as respects suicidal cases, I agree to the extent mentioned by Dr. Bell, of not trusting them on a pledge alone; I may also remark that, however much I might be inclined to favour the abolition of restraint, yet as to this class I would oppose the theory, at least, of its complete disuse. But still, I think that both of these principles might advantageously modify the treatment pursued in regard to the suicidal; for example, at least in the day-time, though not so apparently, being really watched, we might, with benefit release them from restraint, whilst they were placed carefully and pointedly on the pledge not to make any attempt at self-destruction.

Throwing out of consideration suicidal patients, and also the extremely violent, comprehending in asylums where there are no adjunctive poor-houses or other analogous receptacles, but a moderate class, there are left amongst male patients a very large proportionate number, whose liberties I am satisfied might be greatly increased. The experience of the Eastern Lunatic Asylum of Virginia, with which I am familiar, abundantly testifies to such a conclusion. But on investigation, facts of a striking nature in this relation are to be derived from the records of various other institutions.

The idea presents itself, in the first place, in the history of a large number of patients, who, unfortunately for themselves, it is true, are retained at home

* Read at a meeting of the American Association of Medical Officers of Hospitals for the Insane, by Dr. J. M. Galt, physician to the Eastern Lunatic Asylum, Virginia.

a considerable period before sending them to an asylum, or who reside permanently with their friends. Now, although there may be in the annals of every asylum numerous cases where, previously to his reception in the ultimate refuge from the world's wretchedness, the poor patient was chained down in loathsome dens for years and years; and although, perhaps, circumstances of this, as much as those of any other character, have led to the establishment of hospitals for the insane—circumstances, too, which have induced the public to continue for them a generous support; yet still there is a considerable body of sufferers brought to our institutions whose previous history exhibits them to have gone about for years, unrestrained, in the neighbourhood of their homes.* Hence the deduction is plain, could not the same liberty be allowed them at an asylum, particularly with the systematic action pursued there, not only as to those but as to all other patients?

But, again, take the example of the village of Gheel. Here we find a theatre on which the experiment proposed has been tried for centuries. Here we have patients traversing the streets, partaking in the social amusements of the inhabitants, going about wholly unregarded, and indeed upon precisely the same footing as ordinary citizens; and that, too, not in small numbers, but with few exceptions all have these privileges. This is, indeed, advancing the system far beyond the point which we advocate, and properly considered, it must, in fact, completely close the discussion. Now, opposing arguments, however, may be alleged in this connexion. First, it may be said that the peasants treat their patients kindly at Gheel, that the children of the commune do not annoy them, and that the whole neighbourhood adapts itself to this state of things. Granted. But any difficulty of this kind might be met by invoking the patient's self-control and self-respect, and by invariably casting the blame of any difficulty whatsoever, not on the public, but on him. I speak from experience when I say, that this plan will prevent any disturbance between the insane and the population external to an asylum. Secondly, it may be urged, that there exists some intention in Belgium to change the system at Gheel to the ordinary one usual in hospitals. But this does not materially influence the question in debate. From the accounts of travellers, in the plan adopted at Gheel there are doubtless many deficiencies; for example, the want of some central power, having entire authority over the whole arrangement. Nor would I be in favour of this exact general outline; I merely bring forward the treatment here pursued, as illustrating the *truth*, fixed and immutable, in my opinion, at least, as illustrating it most conclusively, that the patients in our asylums have not a degree of liberty allowed them which they could enjoy without the least disadvantage either to themselves or the public. And if this be so, there are none who can possibly wish to deprive them of any liberty which would lead to no injurious consequences, no matter whether they approve or disapprove of the details of the plan in operation in Belgium.

Another circumstance, leading to the conclusion to which we have arrived, is found in a work published by M. Scipion Pinel in 1844, entitled, "Traité de Pathologie Cerebrale." He therein mentions an extraordinary experiment that was tried at the Salpêtrière, which, it is almost needless to remark, contains a large number of patients labouring under mental alienation of long standing; seventy-two of these old cases, looked upon as incurable for years, were sent back again into the world, and only three ever returned. He supposes that there are many chronic cases in establishments for the insane, who, from indolence, from the fear of giving up a life of tranquil ease for one of labour, shrink from recovery; who, from finding themselves amongst

* Probably, indeed, many persons retain recollection of having, in their boyish thoughtlessness, worried and played tricks on these helpless creatures.

the insane, continue to consider themselves as such; and thus remain as inmates merely from these circumstances, and being, as it were, not really deranged. With his explanation, however, or the point—how far they are of unsound mind—we have nothing to do. We would simply mention the success of this experiment with the chronic insane, patients in whom, at any rate, aught like excitement or combativeness has long ago disappeared; and from the result at the Salpêtrière, we would immediately deduce the inference, that to many of the inmates of our asylums might be very suitably granted the minor liberty of going about at will in the neighbourhood of the institution in which they resided.

Another argument which may be urged, is the fact that, in various asylums, we find mention of such liberty as we propose being entrusted to a few of the inmates; for the same liberty might be easily extended to a greater number. Here, as elsewhere, we should never forget the principle contained in any measure; the moment we detect a principle to be involved, and admit its existence, we then establish a rule, that applies not only to a few, but to many. To give an example, Dr. Webster, in his valuable "Notes on Provincial Asylums for the Insane in France,"* remarks, concerning the asylum at Armentière:—"Although no farm is attached to this institution, the gardens adjoining afford means for employing some of the inmates in out-door work. In addition to such occupations, small gangs of patients, under the charge of attendants, are permitted to labour in the fields belonging to the townspeople, from whence they always return to dine in the asylum, but again resume work in the afternoon. For this employment, each patient receives a gratuity of twenty-five centimes per day, which is appropriated to form a fund for after benefits, or occasionally to augment their present comforts. Various inmates are likewise allowed to leave the asylum, under similar regulations, to work for persons in town; some as masons, and others to dig the foundations of new houses now in course of construction. This privilege is appreciated by the poor lunatics, and seems to be beneficial." Again, Dr. Cumming, in his "Notes on Lunatic Asylums," &c., observes, concerning the asylum at Gronnestein, in Germany, that many of the able-bodied patients of the institution work with an attendant on the lands of neighbouring farmers. And, speaking of the asylum for the East Riding of Yorkshire, he states that "Mr. Hill, the able superintendent, even sends parties, of both sexes, to market with vegetables raised on the grounds of the asylum; and the city of York is thus partially supplied by lunatic labour." So Dr. Wilson observes, in the "Report of the Bloomingdale Asylum for 1842":—"Most of the patients take frequent and extensive walks under the supervision of an attendant, although many to whom such supervision would be irksome are allowed, when their situation will admit of it, to ramble at their pleasure, upon giving assurance of their return; and but seldom have such promises been broken." Whilst Dr. Awl writes, in 1841, concerning the patients in the Ohio State Asylum:—"Pledges are often successful, without the necessity of personal restraint. We are seldom disappointed in the word of a patient, seriously given, and 'upon honour.' A number of the peaceable and orderly have the entire freedom of the farm upon these terms, and are sometimes sent down to the city."

In the "Report of the Eastern Lunatic Asylum, of Virginia, for the year 1844," it is observed:—"During a period, longer than twelve months, there has been no enclosure around the southern yard of the institution; and thus nearly all the male patients have, in point of fact, had no barrier whatsoever to going wheresoever they pleased during most of the year just passed." The period here referred to may be considered an intermediate point, in which an uncommon

* Psychological Journal.

degree of liberty, gradually increasing for a year or two before, now reached a maximum, and continued at this maximum for some years succeeding that period. A large number of the inmates, under this absence of restriction, were permitted to ramble into the adjacent country, wheresoever they pleased, unattended, except into town, where it was otherwise forbidden them to go. They would thus take excursions in the woods and fields after nuts and fruit; they would fish in a pond a mile or two off; and bathe in a creek situated at an analogous distance. Going in and out of the wards thus freely, the asylum lost its prison-like appearance. When religious services were held in town, they would also attend them; and, strange to say, when they were of the most exciting character, no harm ensued. During the last Methodist revival that occurred at Williamsburg (in the summer of 1849), three patients attended the meetings very regularly every day, and all of them recovered at that time. The theory of their being permitted to hear preaching, although in town, was that, as an unusual thing, some sane person connected with the asylum would almost always be there. But, in truth, the rule forbidding their entrance into the village was never preserved by them entirely.

This system of extensive liberty was one that was rather the result of time than any sudden action, which was rather the result of gradual experience than any *à priori* theory; and that, moreover, was rather dependent on a varied condition of mind than upon the capacity of each patient enjoying such freedom to give a pledge, and his doing so—although we have headed our article with this title, for the sake of convenience, and remarks into which we would be almost necessarily led, and that, moreover, concerned a subject of importance in the management of the insane. The circumstances leading to one general effect were so various, that it would be difficult to point out the exact operation of each; although, as in other matters, results may be here systematized and reduced to a scientific hypothesis. It will, perhaps, be sufficient for me to point out the fact conceived of a practical bearing; and in this mode of viewing the question, the patients of the asylum may be divided into several classes. In the first place, there are a number of the insane who have been in the institution for many years, and who are in a state of dementia. For a very long period, these have been taken out daily to work. This was the case even before the still existing arrangement was adopted, of having two of the four wards into which the male department is divided, with the doors unlocked during the day, the gates of the enclosure around the institution being also open. These patients would then industriously pursue their daily avocations, and would never care about going outside the premises. No pledge was required of them, and they were incapable of attending to it if it had been. A few, with more active minds than the remainder, would occasionally venture on a fishing excursion, or into the surrounding woods. With many the same vegetative life is pursued in this respect, as in others where they are not allowed to pass freely into the yard outside the building and courts. They would thus, therefore, proceed regularly to the wood-yard and the garden, and never think of advancing their footsteps into town or beyond the immediate neighbourhood. Monomaniacal patients, on the other hand, constitute a class where the pledge was necessary. So with those having, as a peculiar propensity, the idea of returning home. Thus, likewise, with the convalescent.

Now, the advantages of this system were very patent. The patient, in the first place, was rendered much happier. His health was also improved by being so much in the open air, and the general health of the establishment was promoted by keeping the atmosphere of the wards pure, through the mass of the patients being far away from them during most of the day. The faculties of many which, through disuse, would have sunk into a complete state of imbecility, were preserved from such decline by contact with the world. So again all the evils from the assimilation of an asylum to a prison were dissipated. Moreover, the public, from having constant intercourse with these inmates, had proper ideas conveyed to them as to the management of the asylum, and false reports were warded off; for an immediate reply to any one of these would be, "Why, we should have heard the patients of our acquaintance speak of it." And even when the patient conveyed the opposite impression, the general plan of unrestrained intercourse with the public neutralized every evil influence of such a nature. On the whole we may state that the circumstances attending this system were decidedly of a character embodying the greatest degree of freedom found under recent management of the insane; and the advantages were similar to those following increased freedom everywhere.

In looking over the experience of the period, comprising some years, in which the patients were so unrestricted, we cannot perceive any inherent evils which would counterbalance the attendant benefit. So far, for example, as elopement is concerned, to which a system of the kind might, *à priori*, seem liable, the Eastern Asylum will compare favourably with other institutions. And the unpleasant occurrences consequent were few, of little importance, and need not be detailed—such, for example, as a patient obtaining ardent spirits. Occasionally there was some dissension with boys who molested the patients, but on no occasion was there any serious result. Our inmates were always perfectly aware that any dispute with boys or others, exterior to the asylum, would tend to a curtailment of their privileges, whether in any particular difficulty they were to blame or the reverse.

The system under discussion was terminated by the interference of the grand jury of James City County. Nothing was alleged in this instance against it, except, perhaps, a trifling depredation on vegetables or something of the kind, at one of the adjacent farms. But the judge in his charge alluded to the circumstance, that there existed danger from the simple fact of patients going at large; and he remarked that the mere sight of the insane might have a pernicious effect on ladies. The whole affair I think could be clearly traced, not to the true merits of the question involved, but to an unfortunate disposition existing between the townspeople and the inhabitants of the contiguous counties, alike as to the citizens of the place and its institutions; together with a considerable admixture of political antagonism, feelings of intense excitement in connexion with recent alterations in the government of the asylum, and violent dissensions thereupon in the community. Since this edict of the august body by whom it was enacted, the directors have entrusted the exercise of the male patients to three officers, as their exclusive duty.

The result of the experience elsewhere, in the forms which I have pointed out, and the modification of liberty allowed to the patients in the Eastern Lunatic Asylum, I consider such as should lead to a similar extension of privileges in other institutions. In the first place, I would advise in this regard, that the central authority should be particularly careful that the welfare of the lunatics of a whole state be not sacrificed to the prejudices of a petty locality. And to the same intent, I think that before an asylum is established, it ought to be distinctly understood that the patients of the institution would be allowed a large degree of liberty, and that the people of the vicinity would not be permitted to interfere with this question. If evils were to be removed, let the central authority inquire into them, and take the necessary steps. It should be understood that the true view of this question was such, as I may be allowed to express myself, in the phraseology of a patient in the Eastern Lunatic Asylum, to the effect—"That the people of Williamsburg thought the asylum was very near them, but that it was the city of Williamsburg that was very near the asylum." Secondly, one disadvantage under which the Eastern Lunatic Asylum laboured, from its situation, should be, and with care in general might be, avoided, that is, there should be only one entrance to the demesnes of the establishment. Then the officer of the gate-house has entire control of the inmates generally.

Thirdly, I think the plan of confinement in asylums ought always to be modified by placing some of the patients under charge of its officers, to board in the neighbourhood, and particularly insane artisans. The last-mentioned class I would place with persons of their trade in the vicinity, leaving, of course, their supervision to the superintendent of the institution. On the topic just alluded to, I have no time here to enter at large, but I am satisfied that, by an arrangement of the kind, we could frequently act beneficially upon certain inmates, on whom the resources of an asylum had previously been lavished in vain; whilst, by retaining the supervision of a competent medical man, a new set of arrangements would thus be within our power, without the prominent deficiency so peculiarly felt in the cases of patients under private treatment. By such a procedure, we would also create in the vicinity of an institution, for those afflicted with mental alienation, an order of persons familiar with the management of lunacy.

---

